# Supplementary material for: Detecting LLM-generated peer reviews
Source: PLoS One. 2025 Sep 22;20(9):e0331871. doi: 10.1371/journal.pone.0331871 (PMC12453209; doi:10.1371/journal.pone.0331871)
Supplement: S2 Appendix — (PDF) [file pone.0331871.s002.pdf]

S2 Appendix. Greedy coordinate gradient algorithm.

In this section, we describe how the Greedy Coordinate Gradient (GCG) algorithm is adapted to our setting. An LLM can be formally represented as a conditional distribution denoting the probability of generating the next token given the sequence of preceding tokens. Mathematically, an LLM is defined as:

$$\mathsf{LLM}(x_1, x_2, \ldots, x_i) = P(\,\cdot \mid x_1, x_2, \ldots, x_i),$$

where $(x_1, x_2, \ldots, x_i)$ denotes the sequence of tokens generated up to time step i, and P represents the probability distribution over the possible choices for the $(i+1)$-th token, conditioned on the prior i tokens.

In our setting, the input sequence $(x_1, x_2, \ldots, x_i)$ consists of the system prompt SYS, the abstract to review ABS, along with the GCG-optimized text sequence $\mathsf{OPT} = (o_1, o_2, \ldots, o_t)$ appended, and the user prompt USR. Let $W = (w_1, w_2, \ldots w_n)$ represent the tokens of the watermark string. Our objective is to maximize the probability that the LLM starts its response with the watermark token sequence W, which can be formalized as follows:

$$\underset{(o_1,o_2,\ldots,o_t)}{\text{maximize}}\; P(w_1, w_2, \ldots w_n \mid \mathsf{SYS}, \mathsf{USR}, \mathsf{ABS} + \mathsf{OPT})$$

$$\underset{(o_1,o_2,\ldots,o_t)}{\text{maximize}}\; \prod_{j=1}^{n} P(w_j \mid \mathsf{SYS}, \mathsf{USR}, \mathsf{ABS} + \mathsf{OPT}, w_1, w_2, \ldots, w_{j-1}).$$

A standard approach to achieve the above objective is to minimize the negative log-likelihood loss of the watermark tokens with respect to the optimizable tokens, i.e.,

$$\underset{(o_1,o_2,\ldots,o_t)}{\text{minimize}}\; -\sum_{j=1}^{n} \log P(w_j \mid \mathsf{SYS}, \mathsf{USR}, \mathsf{ABS} + \mathsf{OPT}, w_1, w_2, \ldots, w_{j-1}).$$

The GCG algorithm begins by initializing the optimizable tokens in OPT as a sequence of placeholder tokens (‘*’). Then, it optimizes these tokens in an iterative fashion. In each step, it randomly selects a token o_i and replaces it with one of the top k tokens for location i with the highest gradient values of the above loss function. It generates multiple such candidate token sequences and picks the one that minimizes the loss function. In our experiments, we set $k = 256$ for choosing the top-k tokens during token selection and the number of candidate sequences to 512 in each iteration.
