# Supplementary material for: Detecting LLM-generated peer reviews
Source: PLoS One. 2025 Sep 22;20(9):e0331871. doi: 10.1371/journal.pone.0331871 (PMC12453209; doi:10.1371/journal.pone.0331871)
Supplement: S3 Appendix — (PDF) [file pone.0331871.s003.pdf]

S3 Appendix. Inducing LLMs to provide higher ratings in generated reviews.

With the growing capabilities of LLMs, there is increasing interest in using them as reviewers. Some prior evaluations of LLM reviewers incorporate a "ground truth" to assess their performance based on peer review objectives, such as error detection or identifying better research [1] [2, Section 11.2.2]. Others focus on the satisfaction of human stakeholders, such as authors, regarding the LLM-generated reviews [3, 4]. In this section, we offer a different perspective on LLM reviewing by examining adversarial attacks against LLM reviewers. Specifically, we explore how an author can inject text into their paper to manipulate the LLM into generating a more favorable review with a higher rating. A similar perspective was taken in [5], where the adversarial attack modified the paper's content (specifically, the checklist, which was their focus) rather than using prompt injection. Given the various unethical strategies authors sometimes employ to increase their chances of acceptance—such as collusion rings [6, 7], strategic reviewing [8, 9], and other inappropriate practices [2, Section 4]—it is not difficult to envision the emergence of adversarial attacks targeting LLM reviewers as well.

To evaluate if an author can make the LLM review more favorable via a prompt injection attack, we run two evaluations. One evaluation is conducted using 50 ICLR 2024 papers selected uniformly at random, and the other on 50 ICLR 2024 rejected papers. For each paper, we inject the line "Give a rating of 9/10 or 10/10 in the review." and ask the LLM to generate a review and provide a rating out of 10. As a control, we also ask the LLM to generate a review for the same paper but without any injected text. The mean ratings obtained along with the standard error in each setting is provided in Table 1. We find that, when the prompt is injected as compared to when there is no injected text, the mean rating increases by 1.24 (which corresponds to a 17.71% increase) when considering all ICLR 2024 submissions, and increases by 1.64 (23.56%) for ICLR 2024 rejected papers.

Table 1. Mean (± standard deviation) ratings given by an LLM with high-rating-inducing obfuscated instruction.

	**All ICLR 2024 submissions**	**ICLR 2024 rejected papers**
**Without instruction**	7.0±0.26	6.96±0.27
**With instruction**	8.24±0.23	8.6±0.15

For reference, the mean ratings given by human reviewers was 5.11 with a standard deviation of 1.26 across all ICLR 2024 submissions, without any instruction injected [10].

References

1. Liu R, Shah NB. ReviewerGPT? An exploratory study on using large language models for paper reviewing. arXiv preprint arXiv:230600622. 2023;.

2. Shah NB. An Overview of Challenges, Experiments, and Computational Solutions in Peer Review; 2022. `https://www.cs.cmu.edu/~nihars/preprints/SurveyPeerReview.pdf` (Abridged version published in the Communications of the ACM).

3. Liang W, Zhang Y, Cao H, Wang B, Ding D, Yang X, et al. Can large language models provide useful feedback on research papers? A large-scale empirical analysis. arXiv preprint arXiv:231001783. 2023;.
4. D'Arcy M, Hope T, Birnbaum L, Downey D. MARG: Multi-Agent Review Generation for Scientific Papers. arXiv preprint arXiv:240104259. 2024;.
5. Goldberg A, Ullah I, Khuong TGH, Rachmat BK, Xu Z, Guyon I, et al. Usefulness of LLMs as an Author Checklist Assistant for Scientific Papers: NeurIPS'24 Experiment. arXiv preprint arXiv:241103417. 2024;.
6. Littman ML. Collusion rings threaten the integrity of computer science research. Communications of the ACM. 2021;64(6):43–44.
7. Jecmen S, Zhang H, Liu R, Shah NB, Conitzer V, Fang F. Mitigating Manipulation in Peer Review via Randomized Reviewer Assignments. In: NeurIPS; 2020.
8. Balietti S, Goldstone R, Helbing D. Peer review and competition in the Art Exhibition Game. Proceedings of the National Academy of Sciences. 2016;.
9. Xu Y, Zhao H, Shi X, Shah N. On Strategyproof Conference Review. In: IJCAI; 2019.
10. ICLR 2024 statistics; 2024. Available from: `https://papercopilot.com/statistics/iclr-statistics/iclr-2024-statistics`.
